# From summative MAAS Global to formative MAAS 2.0 – a workshop report

**DOI:** 10.3205/zma001591

**Published:** 2023-02-15

**Authors:** Tjorven Stamer, Geurt Essers, Jost Steinhäuser, Kristina Flägel

**Affiliations:** 1 University Hospital Schleswig-Holstein, Campus Lübeck, Institute of Family Medicine, Lübeck, Germany; 2 Network of General Practice Training Institutes in the Netherlands, Utrecht, The Netherlands

**Keywords:** MAAS Global, MAAS 2.0, medicine, communication, doctor patient communication

## Abstract

**Background:**

The MAAS Global (*Maastricht History-taking and Advice Scoring List*) is an internationally widely-used instrument in under- and postgraduate medical education. The focus is on the assessment of medical communication and clinical skills. The assessment tool, which has also been used in Germany since 2015, has a high-quality design (e.g. comprehensible structure, appropriate complexity), good psychometric properties and is very user-friendly. An update of MAAS Global, MAAS 2.0, was published in March 2021 with a new and greater focus on context and the formative.

**Method:**

The revised version of the MAAS 2.0 assessment sheet was translated into German with the authors’ permission. Open questions were discussed with the revision process project manager.

**Results:**

The revision was carried out with a view to focusing on the patient’s frame of reference, removing ambiguities identified previously while using MAAS Global, and closer alignment with the underlying Calgary-Cambridge model. Furthermore, the scale used for the evaluation was modified and now uses a formative evaluation range without grade-related classification.

**Conclusion:**

With the content reorientation of MAAS Global to MAAS 2.0, and the associated focus on frame of reference, context, the formative, the revision presented here sets new priorities for future evaluations in the context of under- and postgraduate medical education and the assessment of medical patient communication in general.

## Zusammenfassung

**Hintergrund:**

Der MAAS-Global (*Maastricht history-taking and advise scoring list*) ist ein international vielfältig in der medizinischen Aus- und Weiterbildung eingesetztes Instrument. Schwerpunkte liegen auf der Beurteilung ärztlicher Kommunikations- sowie klinischer Fertigkeiten. Das seit 2015 auch in Deutschland angewandte Beurteilungsinstrument besitzt eine hohe Qualität in Design (u. a. nachvollziehbarer Aufbau, angemessene Komplexität), gute psychometrische Eigenschaften und eine hohe Benutzerfreundlichkeit. Seit März 2021 existiert eine Aktualisierung des MAAS-Global: der MAAS 2.0, mit einer inhaltlichen Neuausrichtung auf Kontext und einem höheren Fokus auf das Formative.

**Methode:**

Die überarbeitete Version des MAAS-2.0-Bewertungsbogens wurde nach Einholen der Erlaubnis der Autoren ins Deutsche übersetzt. Offene Fragen wurden mit dem Projektleiter des Überarbeitungsprozesses diskutiert.

**Ergebnisse:**

Die Überarbeitung erfolgte in Hinblick auf die Fokussierung auf den Bezugsrahmen des/der Patient*in, auf die Bereinigung von in der Vergangenheit ermittelten Unklarheiten bei der Bewertung mittels des MAAS-Globals sowie auf eine stärkere Angleichung an das zugrundeliegende Calgary-Cambridge-Modell. Des Weiteren erfuhr die zur Evaluation genutzte Skala eine Modifikation und nutzt nun eine formative Bewertungsreichweite ohne notenbezogene Einteilung.

**Schlussfolgerung:**

Mit der inhaltlichen Neuausrichtung vom MAAS-Global hin zum MAAS 2.0, und dem damit einhergehenden Fokus auf Bezugsrahmen, Kontext und das Formative, legt die hier vorgestellte Revision neue Schwerpunkte für zukünftige Evaluationen im Rahmen der Aus- und Weiterbildung und der Bewertung von Arzt-Patient-Kommunikation allgemein vor.

## 1. Introduction

Successful communication between doctors and patients has a positive effect on patient satisfaction, participatory decision-making and treatment adherence [1], [2], [3], [4], [5]. It is of critical importance that good communication skills – i.e. skills that enable the physician to communicate competently with the patient – can be learned [6]. While there is progress in the field of doctor-patient communication optimization within under- and postgraduate medical education in Germany [7], [8], communication training for doctors in postgraduate medical education, for example during specialization in general medicine, have been an integral part of postgraduate education in the Netherlands for over 40 years [9], [10], [11]. MAAS Global (*Maastricht History-taking and Advice Scoring List*) [12], which is seen as very good in terms of structure and design, good to very good regarding psychometric properties and as being very user-friendly, is used as an assessment tool [13], [14]. In the Netherlands, MAAS Global is used as an instrument in both under- and postgraduate medical education. The German version is also used in medical degree courses and postgraduate education [6]. In addition to MAAS Global, various other assessment instruments for evaluating communication skills are available in Germany. One of the established scales is the Calgary-Cambridge Guide, which allows for a detailed assessment of doctor-patient consultation with 71 items in seven sections, ranging from the introduction to the structure of a consultation, to exploration and the completion of a consultation [15]. The Frankfurt Observer Checklist for Communication (FrOCK) contains 31 items and evaluates the start and end of a consultation, the structure, listening, appropriate language, non-verbal communication and the communicative atmosphere. The evaluation is based on German school grades (1=best, 6=worst) [16]. Another available tool is the Cologne Evaluation Form for Communication (KEK), containing 25 items. This serves as an OSCE checklist and addresses factors such as relationship building, listening to the patient’s concerns as well as exploration and participatory decision-making [17]. The Berlin Global Rating system is also available as an evaluation tool for communication skills. 

On a 5-point Likert scale spread over four items, this instrument allows for the consideration of patients’ feelings and needs, focuses on the structure of the consultation and considers verbal and non-verbal expression during the consultation [18]. MAAS Global differs from the assessment instruments listed here in that not “only” communication skills but also medical skills can be assessed in the course of the consultation. For this reason, this instrument makes it possible to give targeted individual feedback. This creates the possibility of a holistic view of the skills that are required for a successful consultation.

### 1.1. From MAAS Global to MAAS 2.0

MAAS Global focuses on the evaluation of medical communication and clinical skills. It is divided into two main communication parts. The first section assesses communication skills for each phase of the consultation, and the second section assesses general communication skills. A third section in respect to the evaluation of clinical skills completes the instrument.

With the advent of MAAS 2.0 in 2021, MAAS Global-D [5], [19] which has also been used as an assessment tool in Germany since 2015, was updated. The focus of MAAS Global was on the summative assessment of doctors and less on the process of further developing communication skills. Having used the tool over a number of years, it was concluded that the assessment guidelines of MAAS Global needed revision, culminating in the MAAS 2.0 modifications. The descriptions of some items have been adjusted and some behavior criteria have been rewritten. The corresponding explanations in the MAAS 2.0 manual have also been revised and additional details have been added for extra clarity and bring the context of the patient consultation to the fore ([https://www.huisartsopleiding.nl/toetsen-beoordelen/maas%20-2-0/], last accessed on 15/12/2022). Simultaneously, more detail was added to the associated manual and the assessment guidance expanded. The predecessor of MAAS 2.0, MAAS Global, contained references to the competencies that are described in the CanMEDS roles [20] and is based on the counseling model of the Calgary-Cambridge approach. The latter is an internationally established method for teaching and training clinical communication skills [21]. The new version of the Dutch assessment instrument, MAAS 2.0, in particular aims to emphasize the Calgary-Cambridge scheme as the underlying model. This concept, created by Kurtz and Silverman in 1996, consists of a five-step guide which, if followed, should facilitate successful doctor-patient communication. Furthermore, its approach focuses on constructive structuring of consultations and on building relationships during consultations [22]. Also new is the increased focus on the context of the consultation to be assessed, higher weighting of consideration and evaluation of partial aspects, and the emphasis on the aspect of shared decision-making in the context of doctor-patient communication [23]. Overall, the changes from MAAS Global to MAAS 2.0 constitute a change from a summative to a formative assessment format.

The project report presented here describes the German translation of MAAS 2.0 and picks up on the changes from its predecessor, MAAS Global-D ([http://www.uksh.de/allgemeinmedizin-luebeck/Downloads.html], last accessed on 15/12/2022).

## 2. Project description

The revised version of the MAAS 2.0 assessment sheet was translated into German after obtaining the authors’ permission.

The reasons for why MAAS Global needed revision was researched on the website of the Dutch organization for Education and Training of General Practitioners ([https://www.huisartsopleiding.nl/toetsen-beoordelen/maas%20-2-0/], last accessed: 12/10/2022). The first author, who speaks Dutch, viewed the documents available on the website. Open questions were discussed with the project manager of the revision process and co-author (GE). Based on the criteria of the ISPOR Task Force for Translation and Cultural Adaptation [24], the translation process into German consisted of the following steps:

1. obtain permission from Geurt Essers,
2. creation of two independent translations by the project team of the Institute for Family Medicine, Lübeck,
3. development of a consensus version as well as cultural adaptation,
4. approval of the consensus version by Geurt Essers (due to his knowledge of the German language, the back-translation step was omitted).

## 3. Results

The translation was culturally adapted: The term “Beratungsanlass” (cause for consultation) as a translation of the term “hulpvraag” was discussed with the Dutch project manager Geurt Essers. Based on the discussions, the team of authors concluded to replace “Beratungsanlass” (cause for consultation) with “Beratungsursache” (reason for consultation). This term is based on the work of Braun [25], Fink et al. [26] as well as Mader and Riedl [27] and represents a more appropriate terminology for the the context of the lived practice within a GP’s surgery: “The reason for the consultation is what brought the patient to the doctor” [25]. In accordance with these changes, both the text and the evaluation form were adjusted and “cause for consultation” was replaced in with “reason for consultation”.

The order of the evaluation scale was also adjusted (as in the previous version [19]), which – in the German version – now ranges from Good (“Gut”) to Poor (“Unbefriedigend”).

The changes made in MAAS 2.0 are presented below for each of its two parts:

1. communication skills for each phase of the consultation and
2. general communication skills.

**Figure 1:**
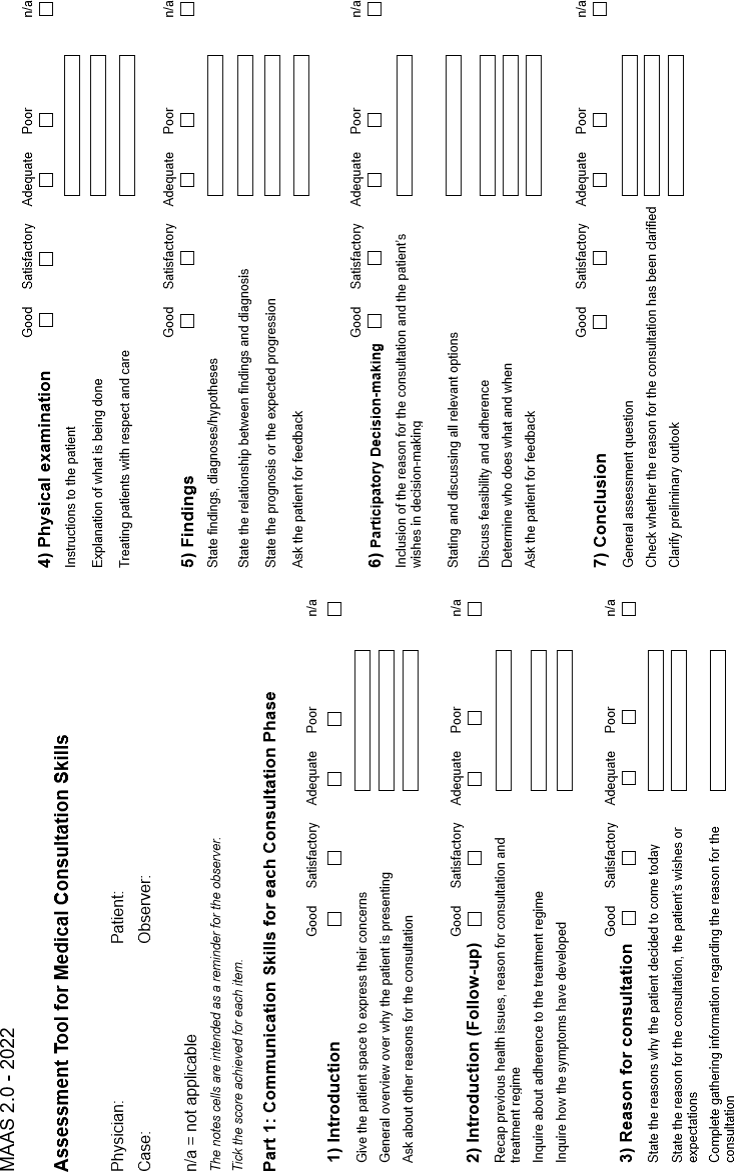
Part 1 of the MAAS 2.0 assessment tool for communication skills for each phase of the consultation

**Figure 2:**
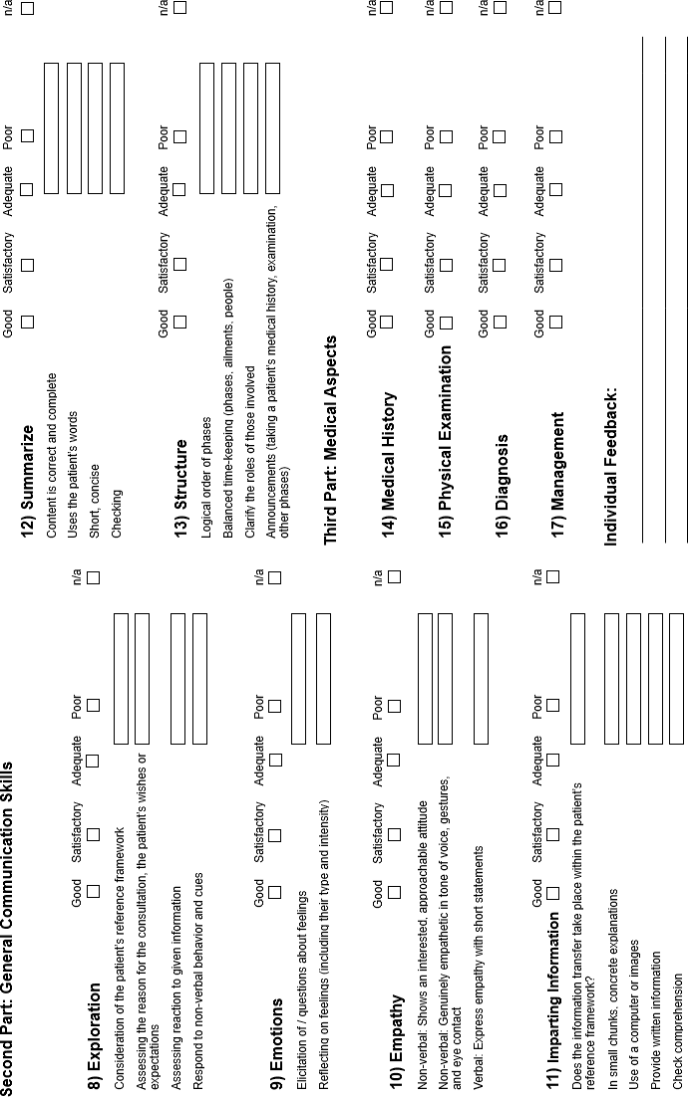
Part 2 of the MAAS 2.0 assessment tool for communication skills

Concrete changes in the content of the first part can be found in figure 1 (Fig. 1). Corresponding modifications of the second part are shown in figure 2 (Fig. 2). The changes to the explanations and wording at item and sub-item level for both parts of the instrument are described below. The same applies to items that were removed as part of the review. 

### 3.1. Concrete content changes

With regard to the sub-items “inquire about adherence to the treatment regime discussed” and “inquire how the symptoms have developed” under “2. Introduction (follow-up appointment)” the German team of authors decided on a different order compared to the Dutch original in order to inquire about the patient’s well-being first before addressing the measures that may not have been successfully implemented. This change in order is based on the recommendations for starting a conversation as part of medical communication [28].

With the introduction of MAAS 2.0, the order of the items in Part 2 has been revised. Therefore, the item “empathy” (MAAS Global item 13; MAAS 2.0 item 10) is now between the items “emotions” (MAAS Global item 9; MAAS 2.0 item 9) and “imparting information” (MAAS Global item 10; MAAS 2.0 item 11). With these changes, MAAS 2.0 is more adapted to the Calgary-Cambridge-Modell [21]. 

The sub-item “sufficiently throughout the entire consultation” was deleted as a sub-item both from the list of sub-items under item “9. Emotions” and from the list of sub-items under item “12. Summarize”. The reason given by those responsible in the Netherlands was that there were some ambiguities in the practical application of using the instrument with regard to the assessment using this sub-item. According to feedback, a clear separation between the assessment of this sub-item and the assessment of the respective overall item (“9. Emotions”, “12. Summarize") was difficult. Under “12. Summarize”, the sub-item “short, concise, in your own words, checking” was also removed to emphasize that the focus of a consultation is not on the physician’s words – which would draw the patient’s narrative into the direct frame of reference of the practitioner – but instead on the patient’s words and their frame of reference. By using the latter, the patient would also feel better heard and understood. The sub-item “announcing, categorizing” was removed due to the infrequent use of announcing and categorizing conversational elements in the consultation observed in the past. “ask what the patient understood” under item “11. Imparting information” was replaced by a broader “checking comprehension”, which does better justice to an open design of doctor-patient communication.

#### 3.2. Concrete changes to wording

Item “6. Management” was renamed “6. Participatory decision-making”. The lack of clarity as to whether “diagnosis” and “management” as communication items of the instrument should be interpreted as medical content rather than elements of the communication process, impeded constructive feedback on communication. To counteract this ambiguity in future assessments, the item was renamed. As part of this renaming, some sub-items were added. The items added are called “inclusion of the reason for the consultation and the patient’s wishes in decision-making” and “stating and discussing all relevant options”. Both sub-items stem from the sub-item “participatory decision-making, alternatives, risks and benefits” which was eliminated from MAAS 2.0. The item “7. Conclusion” is derived from item 7 in MAAS Global, “evaluation of the consultation”. In the past, this item was increasingly interpreted as an invitation to assess the entire consultation, rather than assessing the conclusion of the consultation as such. This aspect was therefore refocused. This change is accompanied by some changes to the wording, which are shown in table 1 (Tab. 1).

In item 10 “empathy” a differentiation between verbal and non-verbal sub-items was introduced. In MAAS 2.0, under item “10. Empathy”, there are therefore two sub-items now which aim at non-verbal empathy and one sub-item which allows the assessment of verbal elements of empathy.

Regarding item “11. Imparting information”, MAAS 2.0 now focuses more on the patient’s frame of reference. As a result of this adjustment, the sub-item “Announcing, categorizing” was removed in favor of the new sub-item “Does the information transfer take place within the patient’s reference framework?”. In item “11. Imparting information”, the new sub-item “use of a computer or images” was added to include information transfer by means of pictorial representation in the context of medical consultations, since this allows patients to memorize information better [23]. Likewise, the sub-items “comprehensible language” and “ask what the patient understood” were removed and replaced by “provide written information” and “check comprehension” in line with the general reorientation.

Under item “12. Summarize” the sub-item “in own words” was changed to “uses the patient’s words” in order to focus on the patient’s frame of reference.

Finally, some adjustments were made to item “13. Structure”. As part of the development of MAAS 2.0, the sub-item “balanced time management” was clarified for better comprehension (“phases, ailments, people”) in order to include the specific aspects of time management in the sub-item. In addition, the sub-item “clarify the roles of those involved” was added, as potential ambiguity in the definition of the roles of those involved would stand in the way of successful communication.

Apart from the specific content-related changes at the item and sub-item level of MAAS Global, there were additional adjustments as a result of the modification of the Dutch assessment instrument. During selection of all items and sub-items, corresponding literature references drawn from existing communication research were included in the MAAS 2.0 handbook. 

#### 3.3. Fundamental innovations

Another significant innovation is the adjustment of the rating scale. MAAS Global still used a summative rating scale ranging from “0=not present”, “6=poor” to “1=excellent” (reverse numbering as part of the cultural adaptation to the situation in Germany [19]), MAAS 2.0 now uses now a formative evaluation range without numerical classification ranging from “good” through to “satisfactory”, “adequate” and “poor”. This represents a 4-point scale. Weighting takes place within the scope of interpretation given by the criterion. For all items or sub-items, both the extent to which the behavior is shown, if at all, and the quality of the behavior must be included in the assessment in order to be able to justify an assessment on the 4-point scale. To be assessed “good”, the required behavior must therefore not only be present, but also confirmed by the quality of what is displayed. In addition, depending on the context in which the consultation takes place, an item or sub-item may or may not be applicable. If a particular item is not the subject of the specific context, that item is noted as “not applicable”. The evaluation scale of MAAS 2.0 therefore does not refer to quantitative evaluation. The reason given for this is the higher focus on the frame of reference and context. 

As part of the feedback process, the instrument helps by giving concrete feedback and information about the physician’s communication. MAAS 2.0 is not a fixed, objective end point or benchmark, but rather serves as a tool for trainers to observe daily reality and classify communication. Furthermore, with the focus the MAAS 2.0 evaluation scale places on context, evaluators are able to pay attention to detail, weight certain aspects differently, and refine overall impressions in specific areas. Based on the feedback received and the related explanations, a physician is able to formulate (new) learning goals.

#### 3.4. Evaluation

The instrument was piloted as part of a train-the-trainer course for physicians with a license for post-graduate training [29]. The MAAS 2.0 pilot therefore took place in the same environment as MAAS Global-D and similarly successful. The participants were immediately able to use the tool. As part of the training course, the same videos were shown and the learners came to the same results compared with previous training courses. In the accompanying evaluation, input using MAAS 2.0 was ranked of 1.4 on a scale from 1 (best) to 6 (worst).

## 4. Discussion

This article discusses the changes from MAAS Global to MAAS 2.0. In addition to linguistic updates, these primarily represent a reorientation from summative to formative assessment. 

Both forms of performance appraisal are associated with different factors of the learning process. Formative assessment is characterized by individualization and acts as a support in the learning process by emphasizing aspects such as feedback, mentoring and the analysis of individual strengths and weaknesses [30]. Summative assessment acts primarily as final overall assessment and provides information on whether certain competencies were acquired or goals achieved [31]. In addition, in summative evaluations, statistical comparison, e.g. in studies, is more feasible. Since the formative type of performance evaluation relies on a high degree of individualization, the question of potentially increased expenditure of time and money respectively arises with the changeover. Due to the precise structure of MAAS 2.0 which leads an assessor through the assessment process, this is not increased. Likewise, the adjustments to the MAAS 2.0 manual include the removal of some ambiguities that have arisen in the past in the context of the evaluation process of MAAS Global. These should further increase clarity with regard to assessment using MAAS 2.0 and reduce ambiguity. MAAS Global-D was used to support feedback in train-the-trainer courses (TTT courses) run by competence centers for postgraduate education in general medicine, e.g. in Schleswig-Holstein [29]. With the MAAS 2.0, physicians with a license for post-graduate training are given explicit instrument for which they can give constructive feedback within the doctor-patient contact of their postgraduate trainees. To what extent the formative form of assessment can be implemented in this scenario will be examined in the future TTT courses.

## 5. Conclusion

The reorientation of the content from MAAS Global to MAAS 2.0 with a focus on frame of reference, context and the formative represents a significant change in the established Dutch assessment instrument. In the course of the establishment of MAAS 2.0, the new focal points could prove to be important key points in the future evaluation of doctor-patient communication in the context of under- and postgraduate medical education.

## Funding

This work was carried out within the context of the LABORATORIUM-project at the University of Luebeck, dealing with the establishment of an AI-based communication learning assistance. Under the project number 16DHBKI075, this work was supported by the German federal-state initiative to promote artificial intelligence in higher education.

## Competing interests

The authors declare that they have no competing interests. 

## Figures and Tables

**Table 1:**
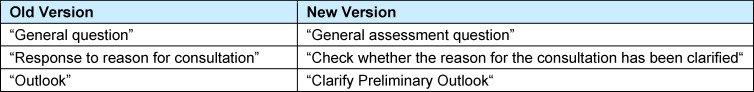
Specific wording changes to the sub-items under item 7 “conclusion”
